# PRESIDENTIAL SYMPOSIUM: EXPANDING THE GEROSCIENCE NETWORK

**DOI:** 10.1093/geroni/igz038.868

**Published:** 2019-11-08

**Authors:** Matt Kaeberlein

**Affiliations:** University of Washington, Seattle, Washington, United States

## Abstract

In keeping with the 2019 GSA Annual Meeting theme of “Strength in Age—Harnessing the Power of Networks”, the Biological Sciences Presidential Symposium focuses on cutting edge approaches to understand the biology of aging using network and systems approaches.

